# The potential benefits of transcranial magnetic stimulation combined with reminiscence therapy on cognitive function in patients with stroke

**DOI:** 10.1515/med-2025-1372

**Published:** 2026-01-13

**Authors:** Roucao Dai, Fengshuang Wang

**Affiliations:** Rehabilitation Treatment Center, Shanghai First Rehabilitation Hospital, Shanghai, China; Organ Rehabilitation Department, Shanghai First Rehabilitation Hospital, Shanghai, China

**Keywords:** potential benefits, transcranial magnetic stimulation, reminiscence therapy, cognitive function, stroke

## Abstract

**Objectives:**

Stroke is a major cause of long-term disability and cognitive impairment, substantially affecting patients’ quality of life and functional independence. Effective strategies for post-stroke cognitive recovery remain limited. To evaluate the effects of transcranial magnetic stimulation (TMS) combined with reminiscence therapy on cognitive function and functional independence in stroke patients.

**Methods:**

A retrospective cohort study was conducted at a rehabilitation hospital from March 2017 to October 2024, including patients with ischemic stroke and cognitive impairment who received either routine treatment (n=68) or TMS combined with reminiscence therapy (n=66). Cognitive and functional outcomes were assessed using the Mini-Mental State Examination (MMSE), Montreal Cognitive Assessment (MoCA), Trail Making Test (TMT), and Functional Independence Measure (FIM).

**Results:**

Post-treatment, the TMS plus reminiscence therapy group showed significantly higher MMSE (27.42 ± 3.08 vs. 25.18 ± 3.41, p=0.0001) and MoCA scores (20.05 ± 3.99 vs. 18.44 ± 4.05, p=0.0020), and shorter TMT times (65.28 ± 9.75 vs. 72.45 ± 10.32, p<0.001). FIM scores were also higher (92.18 ± 7.06 vs. 88.35 ± 7.15, p=0.0024). No significant differences in adverse events were observed.

**Conclusions:**

TMS combined with reminiscence therapy may effectively enhance cognitive function and functional independence in patients with post-stroke cognitive impairment.

## Introduction

Stroke, recognized as a primary contributor to long-term disability and cognitive impairment worldwide, poses significant challenges to patients, caregivers, and healthcare systems [1], 2]. Cognitive deficits following stroke, ranging from mild impairment to overt dementia, greatly affect the quality of life and functional independence of those impacted [3], 4]. Despite advancements in acute stroke management and rehabilitation, addressing cognitive impairment remains a complex and critical aspect of stroke care [5], 6]. Effective interventions targeting cognitive recovery and functional independence were essential to optimize the long-term outcomes and well-being of stroke survivors [7].

Traditional rehabilitation strategies, encompassing physical and cognitive training, have shown some benefit in addressing post-stroke cognitive deficits [8]. However, the quest for innovative and effective interventions to enhance cognitive function and promote neuroplasticity in stroke survivors remains paramount. Transcranial magnetic stimulation (TMS), a non-invasive neuromodulation technique, has attracted attention for its potential to modulate cortical excitability, induce neuroplastic changes, and promote cognitive recovery in various neurological conditions, including stroke [9], 10]. TMS offers a promising avenue for the targeted stimulation of specific brain areas and neural networks implicated in cognitive processes, with the potential to ameliorate the consequences of stroke on brain function [11].

In addition to TMS, complementary interventions such as reminiscence therapy have gained recognition for their potential to enhance cognitive function and emotional well-being in older adults and individuals with cognitive impairment due to stroke [12], 13]. Reminiscence therapy, involving the retrieval and discussion of past memories and experiences, has been associated with positive effects on cognition, mood, and social engagement. By tapping into autobiographical memory and emotional processing, reminiscence therapy offers a unique approach to cognitive stimulation and emotional regulation, potentially synergizing with neurophysiological interventions such as TMS.

The combination of TMS and reminiscence therapy represents an innovative and multidimensional approach to addressing cognitive impairment in stroke survivors. By harnessing the neuroplastic effects of TMS and the cognitive and emotional engagement facilitated by reminiscence therapy, this combined intervention holds the potential to offer comprehensive and tailored rehabilitation for individuals recovering from stroke-related cognitive deficits. However, the specific efficacy and safety of this combined approach in the context of stroke rehabilitation have not been extensively explored.

To address this gap in knowledge, the current study aimed to assess the impact of TMS combined with reminiscence therapy on cognitive function and functional independence in patients with stroke. The findings from this research may facilitate the development of comprehensive and tailored rehabilitation strategies aimed at promoting cognitive recovery and functional independence in this vulnerable patient population. Our hypothesis is that combining TMS with reminiscence therapy will bring greater improvements in cognitive function and functional independence than rehabilitation alone. Compared to a single therapeutic intervention, this combined approach integrates neurophysiological modulation with cognitive-emotional stimulation, potentially producing a synergistic therapeutic effect.

## Materials and methods

### Study population

**Figure 1:**
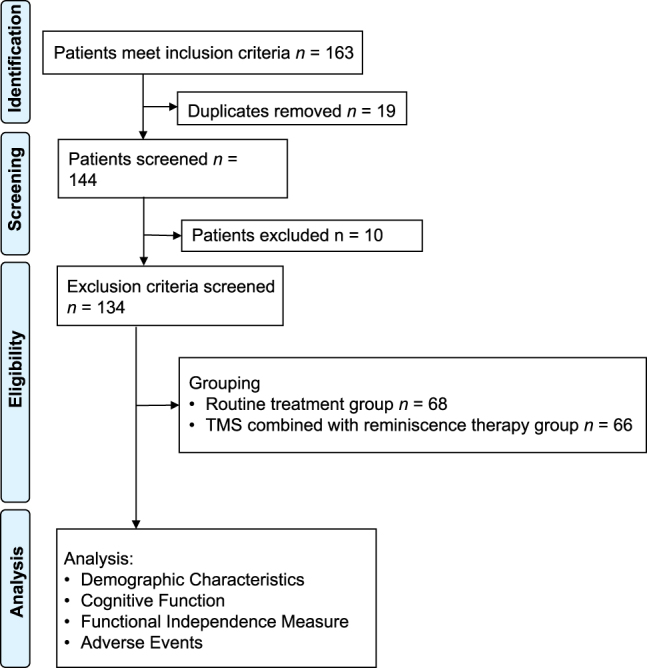
Flow chart of this study.

This study involved a retrospective cohort investigation of stroke patients admitted to our institution from March 2017 to October 2024 and all patients included were divided into routine treatment group (n=68) and TMS combined with reminiscence therapy groups (n=66) based on the treatment methods, resource availability and patient preference (Figure 1). The patient selection process involved multiple steps aimed at ensuring the respect for patient preferences. Initially, physicians provided comprehensive information regarding available treatment options, including the benefits and potential risks, in a clear and comprehensible manner to all patients, ensuring their full understanding of their choices. Subsequently, patients and physicians made joint decisions through honest and open discussions regarding available treatment options, taking into account the patient’s medical condition, personal values, and preferences. Importantly, all patient decisions were made within the framework of medical ethics, ensuring that patient autonomy and informed consent were effectively upheld throughout the decision-making process.

### Inclusion and exclusion criteria

Inclusion criteria: Meet the diagnostic criteria for stroke [14], including both hemorrhagic and ischemic strokes; first-ever stroke; Mini-mental State Examination (MMSE): MMSE Score>17; Montreal cognitive assessment scale (MoCA)<26; normal cognitive functions before stroke; Men or women aged between 18 and 80 years; stable vital signs; Complete medical records.

Exclusion criteria: Patients were diagnosed as having cognitive impairment prior to cerebral infarction; Patients currently taking medications that may interfere with the efficacy assessment of the study therapies, including cholinesterase inhibitors and cytosolic sodium; those with metal or cardiac pacemaker implants; Patients unable to complete assessments during screening process due to severe mental, cognitive, or emotional disorders; Patients suffering from significant diseases affecting internal organs, including the heart, kidneys, and liver; individuals with a history of brain tumors, brain trauma, seizures, or seizure risks. Women who are currently pregnant, planning to become pregnant, or breastfeeding; Patients who were enrolled in other clinical trials pertaining to cognitive impairment within the past month.

### Methods

Routine Treatment Group: Following admission, patients were invited to the rehabilitation center for conventional rehabilitation training, which included body function and cognitive function rehabilitation. Trained physician conducted these sessions once a day for 4 weeks, focusing on daily 30 min of physical motor training (Brunnstrom III-V limb exercises) and 30 min of cognitive training including training on attention (Digit Span progression), calculation, memory (10-item delayed recall), problem solving and executive function (card sorting/clock-drawing).

TMS Combined with Reminiscence therapy Group: The TMS treatment protocol involved using a MagStim Rapid2 device (Rapid2, MagStim, UK) with a figure-of-8 shaped coil, adhering to the manufacturer’s guidelines. The left dorsolateral prefrontal cortex (DLPFC) was targeted at Montreal Neurological Institute (MNI) coordinates (x=−38, y=44, z=26) via individualized MRI-guided neuronavigation (Brainsight v2.4). The resting motor threshold was established by incrementally increasing stimulation to 5 cm lateral to the vertex along the mid-interauricular line. The application level was set at 110 % of the determined motor threshold. Stimulation was applied to the left dorsolateral prefrontal cortex (DLFPC), consisting of 25 consecutive trains delivered at a frequency of 10 Hz, each train comprising 40 pulses and separated by an intertrain interval of 20 s [Basic principles of transcranial magnetic stimulation (TMS) and repetitive TMS (rTMS)]. A total of 20 sessions (20,000 pulses) were administered, at a rate of 1 per day for 20 days. Additionally, patients received reminiscence therapy alongside conventional rehabilitation training. In this program, reminiscence therapy was delivered three times per week in 120-min sessions, structured with the first 60 min devoted to guided reminiscence and the following 60 min to conventional rehabilitation, conducted in groups of 4–6 patients using a standardized 12-chapter reminiscence therapy manual administered by trained therapists. This therapy, conducted thrice a week for 4 weeks, involved 120-min sessions led by trained therapist. The first 60 min were dedicated to reminiscence therapy, covering 12 scheduled chapters designed to evoke memories and foster a comfortable, pleasant environment. The subsequent 60 min were allocated to conventional rehabilitation training. The therapist played an active role in creating an atmosphere conducive to patient-led reminiscence [Reminiscence therapy is a feasible care program for improving cognitive function, anxiety, and depression in recurrent acute ischemic stroke patients: a randomized, controlled study].

### Evaluation indexes

#### General information

General information about the patients, including age, gender, BMI, smoking and drinking history, time since stroke, NIH stroke scale score, presence of hypertension, diabetes, hyperlipidemia, and education status, was systematically retrieved from medical records. Additionally, data on adverse events such as headache, scalp discomfort, and muscle twitching were obtained from the medical records system.

#### Cognitive function assessment

The MMSE is a widely utilized neuropsychological assessment tool designed to evaluate a patient’s intellectual functioning and cognitive impairment, with a maximum possible score of 30 points. Scores ranging from 27 to 30 indicate mild cognitive impairment, scores between 21 and 26 suggest moderate cognitive impairment, while scores from 0 to 20 reflect severe cognitive impairment. The Cronbach’s alpha coefficient for MMSE has been reported as 0.71 [15].

The MoCA scale consists of 11 examination items across 8 cognitive domains, providing a total score of 30 points. Scores of ≥26 are indicative of normal cognitive function, scores ranging from 21 to 25 suggest moderate cognitive impairment, and scores between 0 and 20 reflect severe cognitive impairment. The Cronbach’s alpha coefficient for the MoCA is reported to be 0.87 [16]. Outcome assessors responsible for administering cognitive tests (MMSE/MoCA) and data analysis were blinded to patient group allocation throughout the study.

The Trail Making Test (TMT) assesses processing speed in Part A and executive function in Part B. In Part A, participants are required to connect 25 randomly placed and encircled numbers in the correct sequence, while in Part B, they must connect 25 randomly arranged and encircled numbers and letters in an alternating alphanumeric order. Longer completion times indicate poor performance, and the Cronbach alpha coefficient for TMT was 0.8 [17].

#### Functional independent measurement (FIM)

The FIM is a widely utilized tool for assessing participation following a stroke, encompassing six dimensions of daily functioning: self-care, transfer, sphincter control, communication, locomotion, and social cognition abilities. The FIM consists of 18 items that evaluate the level of assistance required, with scores ranging from 1 (complete dependence) to 7 (complete independence). Consequently, the total score can vary between 18 and 126. The FIM has demonstrated high overall internal consistency, evidenced by a Cronbach’s alpha coefficient of 0.973 [18].

### Statistical analysis

The post hoc analysis was conducted utilizing G*Power 3.1.9.7, selecting the option for “Means: Difference between two independent means (two groups)” based on *t*-tests. The settings included a two-tailed mode, an effect size of d=0.8, and an α error probability of 0.05. After entering the sample sizes for both groups, the power (1-β error probability) was calculated, yielding a power of 0.883. Statistical analyses were performed utilizing SPSS version 29.0 (Chicago, IL, USA). Categorical data were presented as [n (%)], and the chi-square test was applied according to standard criteria when the sample size was≥40 and the theoretical frequency T was ≥5; in this case, the test statistic is denoted by χ^2^. If the sample size met or exceeded 40 but with theoretical frequencies where 1≤T<5, adjustments to the chi-square test were made using appropriate correction formulas. For instances where either the sample size was<40 or theoretical frequency T<1, Fisher’s exact probability method was employed for statistical analysis. Continuous variables underwent normality testing via the Shapiro-Wilk method. For normally distributed continuous data, results are expressed in terms of mean ± standard deviation. Non-normally distributed continuous data and discrete variables were analyzed utilizing the Wilcoxon rank-sum test; these results are presented as [median (25th percentile, 75th percentile)]. A significance level of p<0.05 was deemed statistically significant.

### Ethical statement

This study received approval from the Review Board and Ethics Committee of Shanghai First Rehabilitation Hospital (Approval no. YK-2022-01-012). Informed consent was waived for this retrospective analysis as it exclusively used de-identified data, which posed no potential impact on patient care. This waiver was granted by our Institutional Review Board and Ethics Committee in accordance with regulatory and ethical guidelines governing retrospective research studies.

## Results

### Baseline characteristics

Baseline characteristics of the study participants indicated similar distributions across the Routine Treatment group (n=68) and the TMS Combined with Reminiscence therapy group (n=66) (Table 1). There were no significant differences observed in terms of age, gender distribution, BMI, smoking history, drinking history, time since stroke, NIH Stroke Scale score, MMSE score, hypertension prevalence, diabetes prevalence, hyperlipidemia prevalence, and education situation (p>0.05). These findings indicate that the groups were well-matched at baseline, supporting the comparability of their demographic and clinical characteristics.

**Table 1:** Baseline characteristics of study participants.

Parameters	Routine treatment group (n=68)	TMS combined with reminiscence therapy group (n=66)	t/χ2	p-Value
Age (years)	62.45 ± 7.21	61.97 ± 8.03	0.366	0.715
Gender (male/female)	35 (51.47 %)/33 (48.53 %)	27 (40.91 %)/39 (59.09 %)	1.503	0.220
BMI, kg/m2	25.38 ± 2.14	24.86 ± 1.92	1.460	0.147
Smoking history	19 (27.94 %)	15 (22.73 %)	0.481	0.488
Drinking history	15 (22.06 %)	11 (16.67 %)	0.623	0.430
Time since stroke (months)	10.73 ± 3.12	10.89 ± 3.45	0.276	0.783
NIH stroke scale score	8.72 ± 2.29	8.96 ± 2.14	0.61	0.54
Mini-mental state examination score	23.82 ± 3.26	24.08 ± 3.14	0.45	0.6518
Hypertension	41 (60.29 %)	35 (53.03 %)	0.720	0.396
Diabetes	25 (36.76 %)	21 (31.82 %)	0.364	0.547
Hyperlipidemia	11 (16.18 %)	17 (25.76 %)	1.860	0.173
Education situation			0.717	0.397
– Junior high school and below	43 (63.24 %)	37 (56.06 %)		
– Junior high school or above	25 (36.76 %)	29 (43.94 %)		

### Cognitive function scores

**Figure 2:**
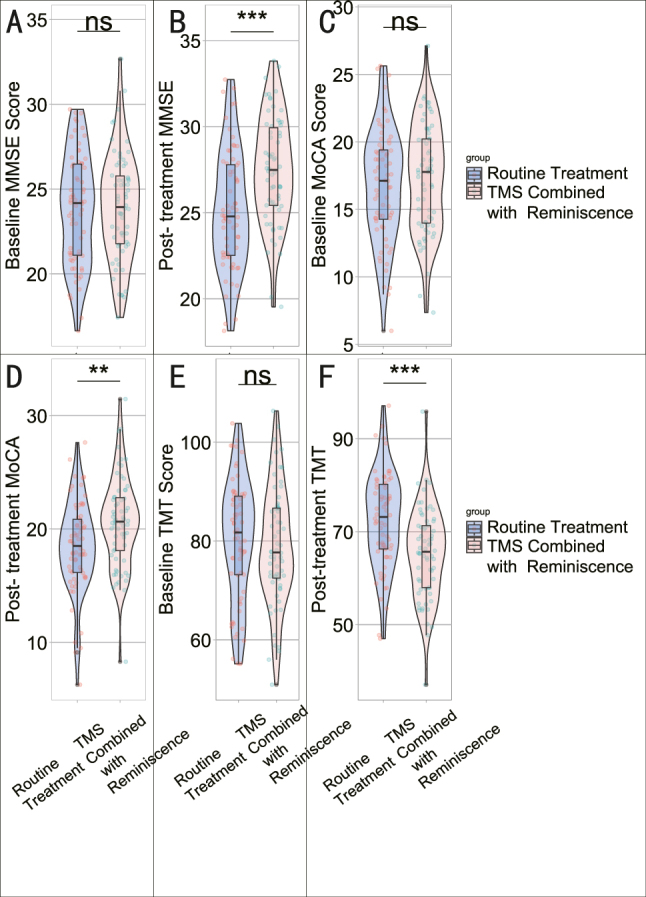
Cognitive function scores at baseline and post-intervention. (A) Baseline MMSE score; (B) post-treatment MMSE score; (C) baseline MoCA score; (D) post-treatment MoCA score; (E) baseline TMT score; (F) post-treatment TMT Score. ns: p>0.05; **: p<0.01; ***: p<0.001.

Comparing the cognitive function scores at baseline and post-intervention between the Routine Treatment group and the TMS Combined with Reminiscence therapy group (Figure 2), the differences in Baseline MMSE Score (23.82 ± 3.26 vs. 24.08 ± 3.14, t=0.45, p=0.6518) and Baseline MoCA Score (16.75 ± 4.16 vs. 17.29 ± 3.99, t=0.76, p=0.4502) were not statistically significant (Table 2). However, post-treatment MMSE (25.18 ± 3.41 vs. 27.42 ± 3.08, t=3.96, p=0.0001), post-treatment MoCA (18.44 ± 4.05 vs. 20.05 ± 3.99, t=3.16, p=0.0020), and post-treatment TMT (72.45 ± 10.32 vs. 65.28 ± 9.75, t=4.134, p<0.001) scores demonstrated statistically significant improvements in the TMS combined with reminiscence therapy group compared to the Routine Treatment group, indicating the favorable impact of the combined intervention on cognitive function.

**Table 2:** Adverse events in routine treatment group and TMS combined with task-specific training group.

Parameter	Routine treatment group (n=68)	TMS combined with reminiscence therapy group (n=66)	χ^2^	p-Value
Headache	11 (16.18 %)	17 (25.76 %)	1.860	0.173
Scalp discomfort	7 (10.29 %)	13 (19.7 %)	2.332	0.127
Muscle twitching	5 (7.35 %)	9 (13.64 %)	1.413	0.235

### FIM scores

**Figure 3:**
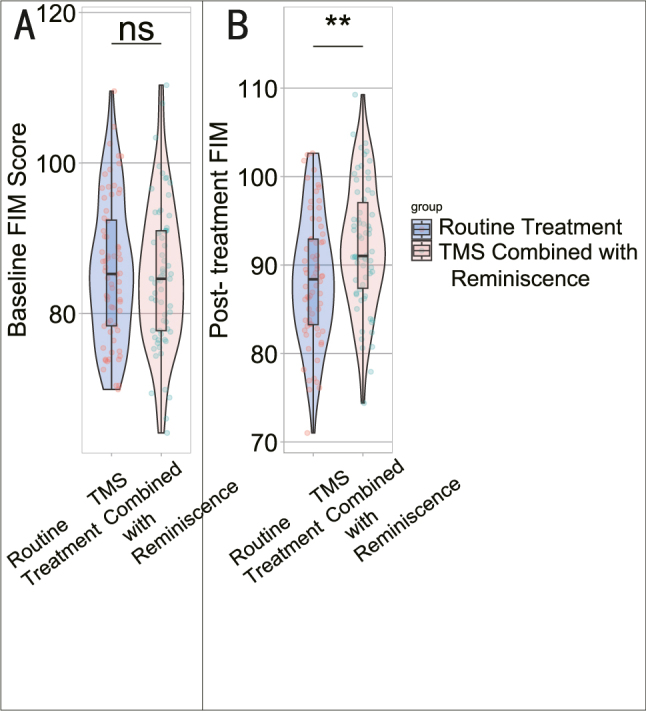
Functional independence measure (FIM) scores at baseline and post-intervention. (A) Baseline FIM score; (B) post-treatment FIM Score. ns: p>0.05; **: p<0.01.

Comparison of the FIM scores at baseline and post-intervention between the Routine Treatment group and the TMS Combined with Reminiscence therapy group (Figure 3) revealed no significant differences in Baseline FIM Score (85.75 ± 9.40 vs. 84.95 ± 9.50, t=0.48, p=0.6296) (Table 3). However, the post-treatment FIM scores showed a statistically significant difference (88.35 ± 7.15 vs. 92.18 ± 7.06, t=3.10, p=0.0024), indicating an improvement in functional independence following the combined intervention. These findings suggest that the TMS combined with reminiscence therapy may have a positive impact on the functional independence of stroke patients.

**Table 3:** Correlation analysis between TMS combined with reminiscential therapy and cognitive function in patients with stroke.

Parameter	r	p-Value
Post- treatment MMSE	0.339	p<0.001
Post- treatment MoCA	0.257	0.003
Post-treatment TMT	−0.351	p<0.001
Post- treatment FIM	0.257	0.003

### Adverse events

The assessment of adverse events in the Routine Treatment group and the TMS Combined with Reminiscence therapy group showed no significant differences in the incidence of headache (16.18 vs. 25.76 %, χ2=1.860, p=0.173), scalp discomfort (10.29 vs. 19.7 %, χ2=2.332, p=0.127), or muscle twitching (7.35 vs. 13.64 %, χ2=1.413, p=0.235) (Table 2). These findings suggest that the combined intervention did not significantly increase the incidence of adverse events compared to routine treatment, suggesting a favorable safety profile for the TMS combined with reminiscence therapy.

### Correlation analysis

The correlation analysis demonstrated statistically significant associations between TMS combined with reminiscential therapy and cognitive function in patients with stroke (Table 3). A positive correlation was identified between the intervention and post-treatment MMSE (r=0.339, p<0.001), as well as post-treatment MoCA scores (r=0.257, p=0.003), indicating that higher utilization of the combined therapy was associated with improved cognitive function. Conversely, a negative correlation was found between the intervention and post-treatment TMT scores (r=−0.351, p<0.001), suggesting that increased utilization of the intervention was linked to decreased time on the TMT. Additionally, a positive correlation was found between the intervention and post-treatment FIM scores (r=0.257, p=0.003), suggesting a potential relationship between the intervention and improved functional independence in these patients. These findings underscore the potential relationship between TMS combined with reminiscential therapy and positive outcomes in cognitive function and functional independence among stroke patients.

## Discussion

Cognitive impairment is a prevalent consequence of stroke, significantly impacting the quality of life and functional independence of affected individuals [19]. The importance of effective interventions to address cognitive deficits in this patient population cannot be overstated [20]. Traditional rehabilitation strategies have shown some benefit, but there remains a need for innovative approaches to further improve cognitive outcomes. In this context, our study focused on evaluating the impact of TMS in conjunction with reminiscence therapy, aiming to elucidate its role in enhancing cognitive function and functional independence among stroke survivors.

The findings of this study demonstrated that the TMS combined with reminiscence therapy group exhibited statistically significant improvements in cognitive function compared to the routine treatment group. Specifically, post-treatment scores on the MMSE, MoCA, and TMT were markedly higher in the TMS combined with reminiscence therapy group, indicating a positive impact on various aspects of cognitive function. These results align with previous research [21], [22], [23] that has highlighted the potential of TMS in enhancing neuroplasticity and promoting cognitive recovery in stroke patients. The addition of reminiscence therapy alongside TMS may have further contributed to the observed improvements, as reminiscence therapy has been associated with positive effects on cognitive function and emotional well-being in older adults and individuals with cognitive impairment [24]. TMS has been recognized for its ability to modulate cortical excitability and induce neuroplastic changes within the brain [25], 26]. It was postulated that the application of TMS may enhance synaptic plasticity, enhance neural network reorganization, and facilitate the recruitment of undamaged brain areas to compensate for the injured regions in stroke patients [27], 28]. By targeting specific cortical areas associated with cognitive processes, TMS may help ameliorate the neurophysiological consequences of stroke and contribute to the restoration of cognitive function [29]. The combination of these interventions appears to offer a promising approach for addressing cognitive deficits in stroke patients, supporting the need for further investigation into their synergistic effects and underlying mechanisms. Furthermore, the addition of reminiscence therapy alongside TMS may tap into the potential of emotional and autobiographical memory recall to enhance cognitive function and well-being in stroke survivors [30], 31]. Reminiscence therapy involves the elicitation of past memories and experiences, which can have meaningful effects on cognitive stimulation, social engagement, and emotional regulation. By promoting the activation of neural networks associated with autobiographical memory and emotional processing, reminiscence therapy may complement the neurophysiological effects of TMS and contribute to the comprehensive rehabilitation of cognitive function. The synergistic effects of TMS-induced neuroplasticity and reminiscence therapy-mediated cognitive and emotional engagement may underlie the observed improvements in cognitive function and functional independence among stroke patients.

Moreover, the findings of this study demonstrated a significant improvement in functional independence as measured by the FIM in the TMS combined with reminiscence therapy group. This finding suggests that the combined intervention not only has the potential to enhance cognitive function but also to translate these improvements into meaningful changes in daily functioning and independence for stroke survivors. This was a critical aspect of stroke rehabilitation, as regaining functional independence was a key goal for patients and was closely linked to their overall quality of life and long-term outcomes [32]. The positive impact of the combined intervention on functional independence underscores its potential relevance in multidisciplinary rehabilitation programs aimed at promoting holistic recovery and long-term well-being for stroke survivors.

The safety profile of the TMS combined with reminiscence therapy was also assessed in this study, with the evaluation of adverse events showing no significant differences between the two treatment groups. This suggests that the combined intervention was well-tolerated and did not significantly elevate the risk of adverse events compared to routine treatment. These findings are consistent with previous research [33,34] indicating the safety and tolerability of TMS in clinical settings, particularly when administered by trained healthcare professionals and in accordance with established protocols. The favorable safety profile of the combined intervention adds to its potential value as a complementary approach to stroke rehabilitation, offering a non-invasive and well-tolerated option for addressing cognitive and functional impairments in this patient population.

The correlation analysis conducted in this study offered further insights into the relationship between the utilization of the combined intervention and its impact on cognitive function and functional independence. The positive correlations observed between the intervention and post-treatment MMSE, MoCA, and FIM scores, as well as the negative correlation with post-treatment TMT scores, highlight the potential dose-response relationship between the intervention and its effects on these outcome measures. These findings suggest that a higher utilization of the combined intervention may be associated with greater improvements in cognitive function and functional independence, supporting the importance of treatment adherence and intensity in achieving meaningful outcomes for stroke patients.

While the findings of this study were promising, several limitations must be considered when interpreting the results. First, the retrospective design of the study introduces inherent biases and limitations related to data collection and confounding variables. Prospective randomized controlled trials are necessary to further validate the safety and efficacy of the combined intervention identified in this study. Additionally, the study sample was restricted to patients from the single institution, which may impact the generalizability of the results to broader stroke populations. Future multi-center research involving larger and more diverse patient cohorts were warranted to confirm the reproducibility of the observed effects and to explore potential variations in treatment responses across different patient populations. Furthermore, future prospective studies with 3–6 month or longer follow-up are needed to evaluate the durability of treatment effects.

Moreover, because randomization was not feasible in this retrospective design and group allocation was influenced by both resource availability and patient preference – although baseline characteristics were statistically comparable – we now explicitly acknowledge that residual confounding may persist and should be addressed in future randomized studies.

The findings of this retrospective cohort study indicate the potential efficacy of TMS combined with reminiscence therapy in enhancing cognitive function and functional independence in stroke patients. The observed improvements in cognitive function and functional independence, along with the favorable safety profile of the combined intervention, support its potential relevance as a complementary approach to stroke rehabilitation. Further research is warranted, including prospective clinical trials and mechanistic studies, to validate these findings, elucidate the underlying mechanisms of action, and explore the broader implications of this combined intervention for stroke survivors. If corroborated by additional evidence, the combined intervention has the potential to contribute significantly to the development of comprehensive and personalized rehabilitation strategies for individuals recovering from stroke, ultimately enhancing their long-term outcomes and quality of life.
